# Supplementary material for: Aging in rural communities: Engagement in indoor leisure activities and older adult health
Source: PLoS One. 2025 Oct 14;20(10):e0334111. doi: 10.1371/journal.pone.0334111 (PMC12520377; doi:10.1371/journal.pone.0334111)
Supplement: Attachment — Submitted filename: Response to reviewers R1.docx [file pone.0334111.s002.docx]

**PLOS ONE**

Ms. No. PONE-D-24-46685 R1

Title: Aging in rural communities: Engagement in indoor leisure activities and older adult health

We sincerely thank the reviewer and the journal office for their detailed comments. We have addressed all the concerns raised by Reviewer 1 and the journal office by revising the manuscript. These revisions include providing more detailed explanations about the selection and assignment of participants, enhancing the discussion, and correcting the manuscript format. We believe these changes have significantly improved the quality of the manuscript. We hope the revised manuscript satisfactorily addresses all issues and is now suitable for publication in PLOS ONE. Below, we provide our point-by-point responses (in blue) to the reviewer’s comments and journal requirements, along with the corresponding page and line numbers in our revised manuscript (PONE-D-24-46685 R1).

**Reviewer 1**

I would like to express my gratitude for the opportunity to review the manuscript titled "Aging in rural communities: Engagement in indoor leisure activities and older adult health." In my view, this type of research, which examines the effectiveness of various physical activities in older adults, is highly relevant. From both a scientific and methodological perspective, I find the manuscript to be rigorous in its justification, design, analysis, and interpretation.

However, there are some areas that require clarification and improvement, which, if addressed, would enhance the overall quality of the manuscript:

**Comment #1** There is a lack of clarity regarding the selection and assignment of participants to each group. A more detailed explanation is needed regarding the criteria and process for categorizing participants into the respective groups (page 12, line 211).

**Response:** Thank you for your valuable feedback. Regarding the selection and assignment of participants, we have clarified the participant selection principles in Lines 201-205. To address your concern about group assignment, participants were assigned based on their respective community locations to minimize travel inconvenience. Specifically, each community carried out only one type of activity: residents from Tungpian Community participated in horticultural activities, residents from Caipu Community engaged in handcraft activities, and residents from Tushi Community took part in baking activities. This approach ensured logistical feasibility and convenience for participants. The detailed explanation has been added in the manuscript (L216-220).

**Comment #2** Additional clarification is required concerning the effects of physical activities on the ability to perform activities of daily living (page 24, lines 439-451).

**Response:** Thank you for your suggestion. Regarding the effects of physical activities on the ability to perform activities of daily living, while our study found no statistically significant improvement in activities of daily living performance among participants engaging in baking activities, this result aligns with existing literature emphasizing the context-specific benefits of physical activities. As noted by Hammell (2013), the impact of physical activities on activities of daily living often depends on the nature, intensity, and relevance of the activity to the participants' daily routines. In our study, baking activities may not have posed novel physical challenges for participants, as many were already familiar with similar tasks in their everyday lives. Consequently, the transferability of physical benefits to activities of daily living performance was limited.

Moreover, the physical effort involved in baking is generally less demanding compared to structured exercise programs designed to enhance strength, balance, and coordination, which are directly linked to improved activities of daily living performance (Ferreira et al., 2012). Future research could explore combining baking activities with additional physical interventions to evaluate their synergistic effects on activities of daily living. The related texts have been added in the manuscript (L452-458).

**Journal Requirements:**

**#1** Please ensure that your manuscript meets PLOS ONE's style requirements, including those for file naming. The PLOS ONE style templates can be found at

<https://journals.plos.org/plosone/s/file?id=wjVg/PLOSOne_formatting_sample_main_body.pdf>

<https://journals.plos.org/plosone/s/file?id=ba62/PLOSOne_formatting_sample_title_authors_affiliations.pdf>

**Response:** Thank you for your guidance and support. The manuscript has been revised to fully comply with the formatting requirements of PLOS ONE.

**#2** Please provide additional details regarding participant consent. In the ethics statement in the Methods and online submission information, please ensure that you have specified (1) whether consent was informed and (2) what type you obtained (for instance, written or verbal, and if verbal, how it was documented and witnessed). If your study included minors, state whether you obtained consent from parents or guardians. If the need for consent was waived by the ethics committee, please include this information.

If you are reporting a retrospective study of medical records or archived samples, please ensure that you have discussed whether all data were fully anonymized before you accessed them and/or whether the IRB or ethics committee waived the requirement for informed consent. If patients provided informed written consent to have data from their medical records used in research, please include this information.

**Response:** The details regarding participant consent have been added to the manuscript. Please refer to Lines 262–266.

**#3** Please note that funding information should not appear in any section or other areas of your manuscript. We will only publish funding information present in the Funding Statement section of the online submission form. Please remove any funding-related text from the manuscript.

**Response:** The funding-related text has been removed from the manuscript.

**#4** Thank you for stating the following financial disclosure:

“This work was supported by the Agency of Rural Development & Soil and Water Conservation, MOA (former Bureau of Soil and Water Conservation, Council of Agriculture, Executive Yuan) of Taiwan. Grant Number: #110-1.1.1-1.1-020(3).”

Please state what role the funders took in the study. If the funders had no role, please state: "The funders had no role in study design, data collection and analysis, decision to publish, or preparation of the manuscript."

If this statement is not correct you must amend it as needed.

Please include this amended Role of Funder statement in your cover letter; we will change the online submission form on your behalf.

**Response:** Revised.

**#5** We note that you have indicated that there are restrictions to data sharing for this study. PLOS only allows data to be available upon request if there are legal or ethical restrictions on sharing data publicly. For more information on unacceptable data access restrictions, please see <http://journals.plos.org/plosone/s/data-availability#loc-unacceptable-data-access-restrictions>

Before we proceed with your manuscript, please address the following prompts:

a) If there are ethical or legal restrictions on sharing a de-identified data set, please explain them in detail (e.g., data contain potentially identifying or sensitive patient information, data are owned by a third-party organization, etc.) and who has imposed them (e.g., a Research Ethics Committee or Institutional Review Board, etc.). Please also provide contact information for a data access committee, ethics committee, or other institutional body to which data requests may be sent.
b) If there are no restrictions, please upload the minimal anonymized data set necessary to replicate your study findings to a stable, public repository and provide us with the relevant URLs, DOIs, or accession numbers. For a list of recommended repositories, please see https://journals.plos.org/plosone/s/recommended-repositories. You also have the option of uploading the data as Supporting Information files, but we would recommend depositing data directly to a data repository if possible.

We will update your Data Availability statement on your behalf to reflect the information you provide.

**Response:** All relevant data are within the manuscript.

**#6** Please ensure that you include a title page within your main document. You should list all authors and all affiliations as per our author instructions and clearly indicate the corresponding author.

**Response:** The title page has been added to the manuscript.

**#7** Your ethics statement should only appear in the Methods section of your manuscript. If your ethics statement is written in any section besides the Methods, please delete it from any other section.

**Response:** Revised.

**#8** Please include captions for your Supporting Information files at the end of your manuscript, and update any in-text citations to match accordingly. Please see our Supporting Information guidelines for more information: <http://journals.plos.org/plosone/s/supporting-information>

**Response:** This manuscript has no relevant Supporting Information files.
