# Supplementary material for: Aging in rural communities: Engagement in indoor leisure activities and older adult health
Source: PLoS One. 2025 Oct 14;20(10):e0334111. doi: 10.1371/journal.pone.0334111 (PMC12520377; doi:10.1371/journal.pone.0334111)
Supplement: Attachment — Submitted filename: Response to reviewers R2.docx [file pone.0334111.s003.docx]

**PLOS ONE**

Ms. No. PONE-D-24-46685 R2

Title: Aging in rural communities: Engagement in indoor leisure activities and older adult health

We sincerely thank the reviewers for their helpful comments. In this revised manuscript, we have addressed all concerns raised by Reviewer 2 and Reviewer 4. We added a Limitation section to explain the possible influence of group assignment based on location. We also corrected some grammar and wording for clarity. In response to Reviewer 4, we added a Wilcoxon signed-rank test to better analyze the IADL data. We believe these changes have improved the quality of the paper and hope it is now suitable for publication in PLOS ONE. Below, we provide point-by-point responses to each comment, with corresponding revisions shown using Track Changes in the revised manuscript (PONE-D-24-46685 R2).

**Reviewer 2**

The revised manuscript is well-written, methodologically sound, and addresses a timely and socially relevant topic—the impact of indoor leisure activities on older adults' physical and mental health during the COVID-19 pandemic in rural Taiwan. The authors present a thoughtful design, appropriate measures, and a clear analysis using repeated-measures ANOVA.

The paper contributes to the literature on aging, leisure therapy, and rural health promotion. However, I recommend minor revisions before acceptance:

**Comment #1** Clarify Group Assignment Strategy: While the authors explain that group assignment was based on location (to reduce participant burden), this introduces a risk of community-level confounding. Although randomization is not claimed, I suggest adding a brief statement in the Limitations section to acknowledge this limitation and its potential influence on between-group comparability.

**Response:** Thank you for your valuable comment. We have added a statement in the Limitations section (L527–L531) to acknowledge the lack of randomization and the potential for community-level confounding due to the group assignment being based on participant location.

**Comment #2** Proofreading for Minor Language Corrections: A few grammatical or typographical edits are needed. For example:

Line 27: “complete self-reported their activities” → should be revised for clarity.

Line 449: “participants were women who were as familiar with baking as with meal preparation” → consider rephrasing for smoother readability.

**Response:** We have revised the sentence to improve readability: “completed self-reported measures regarding their activities of daily living, depression, and mental and social health.” (L28–L29)

Additionally, the sentence on lines 455-456 has been revised to: “Given that most participants were women with comparable experience in baking and meal preparation,”

**Reviewer 3**

The study has significant merit and high relevance for aging in rural communities. so it may be publishable. Paper has insights and it will help policy making in different stakeholder.

**Response:** Thank you for your positive assessment of our paper.

**Reviewer 4**

This manuscript addresses an important and increasingly relevant topic by exploring the impact of indoor leisure activities on successful aging in rural older adults. The authors present an intervention study comparing horticultural, handicraft, and baking activities and report positive effects on depression, physical, mental, and social health outcomes. The study’s focus on an aging rural population and its practical aim of promoting well-being in resource-limited settings are commendable.

**Comment #1** I noted one major weakness in the study, which I believe makes the results unpublishable in their current form. The study uses a pre- and post-intervention design in the same participants, without any control group. This approach is highly vulnerable to confounding factors such as seasonal changes in mood or health. In the absence of control groups (e.g. no-intervention or delayed-intervention groups), the observed effects cannot confidently be attributed to the interventions.

**Response:** Thank you very much for your insightful comment. We agree that the lack of a no-intervention control group is a limitation and may limit causal interpretations. However, we would like to provide further context and justification for our design.

1. This study was conducted during the peak of the COVID-19 pandemic (August–November 2021), when older adults—especially those living in rural communities—faced heightened risks of social isolation, depression, and reduced access to healthcare. Under these conditions, it was extremely difficult to recruit older adults due to health concerns, mobility limitations, and the general anxiety around participation in any group activity. We prioritized participant safety and inclusiveness and believed it would be ethically inappropriate to ask participants to join the study without receiving any intervention, as required in a no-intervention control group.
2. To partially address this limitation, we employed a between-subjects design, where each community was assigned one specific intervention type (horticultural, handicraft, or baking), which allowed for comparative analysis between groups. While not equivalent to a control group, this structure provided an internal benchmark to observe differential effects among interventions. We implemented repeated-measures over five timepoints (T0–T4) for each outcome, allowing us to track longitudinal trends within each group. Repeated-measures designs reduce error variance and provide robust evidence of within-subject changes. They are widely accepted in intervention research when randomization is not feasible (Leon et al., 2011; Paulus et al., 2014) (Please see L513–L525).
3. Theoretical and empirical justification exists for such quasi-experimental designs in real-world health and aging studies, especially during public health emergencies or in vulnerable populations. Several studies in psychological and public health research have argued that carefully designed single-group pre-post studies and non-randomized designs remain valuable and publishable if properly analyzed and transparently reported (Bakker et al., 2014; Shadish et al., 2002).
4. We have added a Limitations section to explicitly acknowledge the absence of a control group and have emphasized the need for future studies to adopt randomized or delayed-intervention designs where ethically and practically feasible (L532–L540).

We hope this explanation clarifies the rationale behind our study design and supports the contribution this research makes to understanding the health impacts of leisure activities for rural older adults during times of social restriction.

- Leon, A. C., Davis, L. L., & Kraemer, H. C. (2011). The role and interpretation of pilot studies in clinical research. *Journal of Psychiatric Research,* 45(5), 626–629. <https://doi.org/10.1016/j.jpsychires.2010.10.008>
- Paulus, J. K., Dahabreh, I. J., Balk, E. M., Avendano, E. E., Lau, J., & Ip, S. (2014). Opportunities and challenges in using studies without a control group in comparative effectiveness reviews. *Research Synthesis Methods,* 5, 152–161. <https://doi.org/10.1002/jrsm.1101>
- Bakker, A. B., Demerouti, E., & Sanz-Vergel, A. I. (2014). Burnout and work engagement: The JD–R approach. *Annual Review of Organizational Psychology and Organizational Behavior,* 1(1), 389–411. <https://doi.org/10.1146/annurev-orgpsych-031413-091235>
- Shadish, W. R., Cook, T. D., & Campbell, D. T. (2002). *Experimental and quasi-experimental designs for generalized causal inference.* Boston: Houghton Mifflin.

**Comment #2** There are also some minor weaknesses in the study, most notably the use of a paired t-test to compare IADL data. A Wilcoxon signed-rank test would be better, since the data would not be expected to be continuous, normally distributed, have equal variances between groups, and are also bounded at 0 and 8.

**Response:** Thank you for your methodological suggestion. Per your suggestion, we have now conducted a Wilcoxon signed-rank test for the IADL data. The Wilcoxon test confirmed the same significant improvements for the horticultural and handicraft activity groups. This change is reflected in the revised Statistical Analysis (L325–L327) and Results sections (L370–L374).

**In summary, we sincerely appreciate the reviewers’ suggestions, and we believe that our manuscript has been substantially improved because of your helpful comments. We hope that we have addressed all of your concerns.**
