# Supplementary material for: Aging in rural communities: Engagement in indoor leisure activities and older adult health
Source: PLoS One. 2025 Oct 14;20(10):e0334111. doi: 10.1371/journal.pone.0334111 (PMC12520377; doi:10.1371/journal.pone.0334111)
Supplement: Attachment — Submitted filename: Response to reviewers R3.docx [file pone.0334111.s004.docx]

**PLOS ONE**

Ms. No. PONE-D-24-46685 R3

Title: Aging in rural communities: Engagement in indoor leisure activities and older adult health

We sincerely thank the reviewers for their helpful comments. In this revised manuscript, we have addressed all concerns raised by Reviewer 4 and the editor. We believe these changes have improved the quality of the paper and hope it is now suitable for publication in PLOS ONE. Below, we provide point-by-point responses to each comment, with corresponding revisions shown using Track Changes in the revised manuscript (PONE-D-24-46685 R3).

**Editor, Dr. Bifeng Zhu,**

**Comment #1** The concerns of the reviewer do not necessarily indicate that the results of the paper are incorrect, but rather that the methodology is flawed. And as mentioned by the reviewer and authors, the flaw is caused by specific events during the pandemic. We cannot avoid (ignore the flaw) or overcome it (supplement the complete control experiment). It is just like the complexity of sociological issues, which is often determined by multiple factors. The continuous contemplation and collision of ideas between the author and the reviewer in pursuit of truth in this paper have high academic contribution and reflect the diversity of sociological issues.

Therefore, I suggest the author to: a) reflect the concerns of the reviewers in the discussion section. b) increase the credibility of the results as much as possible by supplementing necessary tests. c) improve the transparency of data. d) complete legends and labeling.

**Response:** We appreciate the opportunity to revise the manuscript and have addressed the concerns about methodological rigor and transparency. Specifically, we (a) explicitly report multiplicity control and apply Bonferroni adjustment to all pairwise estimated marginal means; (b) clarify in the Results that significance refers to Bonferroni-adjusted p-values. The adjusted results were similar to the original findings, and all major patterns of significance remained unchanged. This demonstrates that our conclusions are not dependent on uncorrected tests and are robust to multiplicity adjustment; (c) provide individual-level data for all key outcome measures as supplementary files; and (c) revise table and figure titles/legends so that they are self-explanatory, with all abbreviations and summary statistics clearly labeled.

**Reviewer 4**

**Comment #1** I would like to thank the authors for revising the manuscript and for adding a detailed rationale for the design, an expanded Limitations section, and relevant citations. I also appreciate the transparent discussion of the ethical and logistical challenges faced during the pandemic. While these additions are helpful, they do not fully address the previously mentioned concerns with the experimental design.

Referring to Paulus et al, one of the additional sources cited in the revised manuscript, a few of the conditions that must be met for a before–after study to yield valid inferences include the following:

- Each participant’s baseline must accurately predict what would have happened without treatment.
- Changes in the environment must be addressed.
- The condition studied should be relatively stable (i.e. not subject to natural recovery or intermittent fluctuations).

While the authors might see some way forward to address these threats to validity, I do not. As these data were collected during the COVID-19 pandemic, a unique and sometimes rapidly changing environment, the unique context likely precludes attempts to address these issues using historical or external data. Indeed, it was implied that some outcome measures (e.g. depression scores) were likely strongly influenced by the environment.

Additionally, the highly confounded study design should effectively preclude the between-group comparative analyses reported here, rather than enabling them as the manuscript states. Prioritizing participant well-being is commendable, but it is my belief that equipoise was neglected in this study design.

I would also like to call attention to several other issues:

Lack of correction for multiple comparisons. The study conducts within-group and between-group tests across multiple metrics and timepoints without any adjustment for Type I error.

Insufficient data transparency. While the authors report summary statistics, reliance on summary statistics alone prevents readers and reviewers from evaluating the validity of the non-parametric analyses. Please provide the underlying individual-level data.

Incomplete legends and labeling. Several tables and figures lack sufficiently detailed legends or labels, making it difficult to interpret the results independently. Reported summary statistics are not clearly labeled, etc. Please ensure that all such materials are fully self-explanatory.

**Response:** We sincerely thank you for the constructive comments. In response, we have:

(1) Reflected reviewer concerns in the Discussion (Lines 413–421), acknowledging the limitations of before–after design under the unstable COVID-19 context (Lines 545–549).
(2) Added Bonferroni correction to reduce Type I error (Lines 328–330, 385–388).
(3) Improved transparency by committing to provide individual-level data as supplementary files.
(4) Revised all table and figure legends to ensure complete labeling and interpretability (Tables 1–3, Figs 2).

**In summary, we sincerely appreciate the reviewers’ suggestions, and we believe that our manuscript has been substantially improved because of your helpful comments. We hope that we have addressed all of your concerns.**
